# Travelling in *Microphis* (Teleostei: Syngnathidae) Otoliths with Two-Dimensional X-ray Fluorescence Maps: Twists and Turns on the Road to Strontium Incorporation

**DOI:** 10.3390/biology13060446

**Published:** 2024-06-18

**Authors:** Clara Lord, Vincent Haÿ, Kadda Medjoubi, Sophie Berland, Philippe Keith

**Affiliations:** 1 UMR 8067, Biologie Des Organismes Et Écosystèmes Aquatiques (BOREA), Sorbonne Université, Muséum National d’Histoire Naturelle, Université de Caen Normandie, Université Des Antilles, CNRS, IRD, CP26, 43 Rue Cuvier, 75005 Paris, France; hvincent75@gmail.com (V.H.); sophie.berland@mnhn.fr (S.B.); philippe.keith@mnhn.fr (P.K.); 2 Synchrotron SOLEIL, 91192 Saint-Aubin, France; kadda.medjoubi@synchrotron-soleil.fr

**Keywords:** diadromous life cycle, environmental transition signature, freshwater pipefish, otoliths, strontium incorporation, synchrotron XRF 2D mapping

## Abstract

**Simple Summary:**

Otoliths, calcified biomineralised structures in bony fish inner ear, are remarkable objects as keepers of individual life, both in terms of time and environment. Otolith microchemical analysis was undertaken with a synchrotron-based scanning X-ray fluorescence 2D high-resolution mapping method and we developed analytical imaging processes to retrieve biological information. Strontium incorporation in the otoliths of four pipefish species from Pacific island streams was analysed, as its presence in otoliths is a sign of life in a marine environment, while its absence corresponds to life in freshwater. Their life cycle is expected to be diadromous, allowing for species sustainability in labile and fragmented habitats. From a biological point of view, we uncovered the existence of plasticity in the diadromous life cycle, enhancing our knowledge of these poorly studied species, which are highly threatened by anthropogenic actions. In addition, our 2D hyperfine-scale images outlined the heterogeneity of strontium distribution in the otolith to a greater extent than what is generally assumed. Our results led us to uncover a trade-off in expression between the biological and chemical driving forces that govern element incorporation in the otoliths. Our methods and results provide a strong basis for future works and prospects in fish biology, ecology, and conservation.

**Abstract:**

Indo-Pacific tropical island streams are home to freshwater pipefish (*Microphis* spp., Syngnathidae). Otoliths were used to uncover life history traits in four species, including a New Caledonian endemic. All four species present the same methodological challenge: their otoliths are small, fragile and mute for growth marks using basic observation tools. Strontium (Sr) is calcium substituent in the mineral lattice, driven by salinity conditions, and thus useful to study diadromous migrations. Synchrotron-based scanning X-ray fluorescence 2D high-resolution mapping allowed us to tackle the global and hyperfine strontium (Sr) distribution. We developed analytical imaging processes to retrieve biological information from otoliths from the data generated via synchrotron analysis. We uncovered plasticity in the life cycle: all species were amphidromous, apart from some freshwater residents from New Caledonia. Understanding life cycle modalities is crucial to categorize species distribution limits and to implement adapted conservation measures, especially when endemic species are at stake. 2D fine-scale images outlined the heterogeneity of Sr distribution: in addition to the trivial Sr incorporation driven by environmental ionic conditions, there is an unusual mosaic arrangement of Sr distribution and we hypothesize that biological control, especially growth during the early life stages, may sometimes overrule stoichiometry. This shows that it is worth studying otolith formation and element integration at imbricated scales, and our methods and results provide a strong basis for future works and prospects in otolith science.

## 1. Introduction

Teleost fish otoliths are paired calcium carbonate structures located in the inner ear. Approximately 98% of the otolith is a CaCO_3_ mineral, usually in the mineral form of aragonite, while the other ~2% is composed of an organic matrix and minor and trace elements [1].

Otoliths have long been used as recorders of individual life as they grow throughout the fish’s life and are not subjected to remodelling [2]. Their incremental growth allows for time estimations; minor and trace elements are preserved in the otolith matrix and their concentrations can reflect environments with different chemical signatures, such as marine or freshwater environments in which the fish has been. These trace elements may also provide information on the physiological state of the fish [3,4].

The study of otolith chemical composition can be used to reconstruct individual migratory and environmental histories (e.g., [5,6,7]). Indeed, Sr concentrations are considered to be stable and much higher in the marine environment. Otolith Sr content is correlated with an ambient Sr concentration in the surrounding water [8]. The strontium/calcium ratio (Sr:Ca) in the otolith, in relation to the transition between water bodies of different salinities, is commonly reported in the range of 2 to 4 mmol·mol^−1^ in freshwater and >6 mmol·mol^−1^ in estuarine and sea water [9,10,11]. This ratio has been commonly used to reconstruct migration patterns in several diadromous species: anadromous salmonid species [9,11], the catadromous genus *Kuhlia* [12], and the eel *Anguilla marmorata*, Quoy and Gaimard, 1824 [13], as well as amphidromous Sicydiinae gobies [5,14].

Unlike other diadromous species, the migration between fresh and marine waters of amphidromous species does not involve reproduction. Adults live, grow and reproduce in freshwater; after hatching, individuals drift downstream to sea where they spend a variable amount of time before returning to rivers [15]. In tropical island rivers, fish are mainly amphidromous (especially Gobiidae and Eleotridae) [16] as the highest likelihood for colonisation of such environments is by organisms coming from the sea and able to tackle the transition between the two biomes [17].

Syngnathidae, including seahorses, seadragons and pipefish, are a charismatic teleost family including about 50 genera and 300 species [18]. They are mainly found in tropical marine areas; however, about 30 pipefish species inhabit freshwater [19] and are essentially distributed in tropical island rivers of the Indo-Pacific. Freshwater pipefish are highly endangered species [20] and their protection is impeded by an important lack of knowledge.

The choice to study freshwater pipefish is not only driven by conservation purposes; it is also motivated by the challenge of studying reluctant otoliths. Pipefish have tricky otoliths: very small in size (<400 µm), fragile and lacking any sort of growth marks using classic observation methods [7].

Nowadays, the most commonly used method is 1D transect or point-by-point methods such as laser ablation coupled to an inductively coupled plasma mass spectrometer (LA-ICPMS). This method has many uses such as in the study of the amphidromous life cycle of Sicydiinae gobies [5], stock assessment of the migratory porcupine fish by Sr marking [21] or the study of the migratory behaviour of commercial fish [22]. With this method, researchers can focus on the early growth region [23] or can obtain life history profiles along a transect over a chosen growth axis of the otolith; these types of transects are often used to detect environmental changes during the individual’s life such as migrations between water masses of varying salinity [5,12,24]. However, interpretation of the results with this powerful but destructive technique (since the laser beam ablates matter leaving a few μm to several tens of μm wide trench on the sample) is carried out under the assumption that the analysed zone chosen is representative of the individual life history. However, caution must be taken since physiological jamming and biomineralization processing may lead to spatial heterogeneity in elemental incorporations [1,10,25]. Adding a second dimension, i.e., 2D mapping of elements, allows refining the study of element incorporation patterns. Elemental 2D maps can be obtained using a UV high-repetition-rate femtosecond laser ablation system coupled to a high-resolution inductively coupled plasma mass spectrometer. This method consists in ablating the entire surface of the otolith repeatedly; each transverse section ablated produces an elemental image [26]. The width of the ablation beam is usually 20–30 μm in diameter. Electron microprobe analysis (EPMA) was also used for otolith analysis. It is fundamentally the same as scanning electronic microscopy, with the added capability of chemical analysis. EPMA allows acquiring precise, quantitative elemental analyses at very small “spot” sizes (c. 1–2 microns), primarily by wavelength-dispersive spectroscopy. But EPMA otolith analysis is usually coupled to LA-ICPMS techniques: the EPMA detection limit of elements is quite high (100 μg/g) and only a few elements in the otolith can be analysed; the LA-ICPMS detection limit is around 0.1–1 μg/g. Researchers use these two complementary techniques, with an overlap of elements that can be measured with both, ensuring the correctness of the results [27,28]. X-ray-fluorescence-based methods have also been developed such as proton-induced energy emission spectroscopy (micro-PIXE) or scanning X-ray fluorescence microscopy (SXFM). These two methods create X-ray fluorescence by exciting atoms either with accelerated protons of high energy (PIXE) or high energy X-rays produced by a synchrotron accelerator (SXFM). At de-excitation, atoms emit X-ray photons characteristic of the element present and the number of X-ray photons emitted gives the quantity present. The size of the spots can vary depending on the technique used and has an impact on element quantification [29]. To analyse elements at the microscale, synchrotron X-ray fluorescence (XRF) is a promising method for producing fine-scale 2D elemental maps and can simultaneously produce multi-element maps of biomineralized structures [7,29,30,31,32]. XRF maps allow studying element incorporation dynamics throughout the otolith at a fine scale (presently down to 0.5 μm) [7,9], even for elements present at low concentrations (lower limit of detection: 1–10 ppm; see [33]).

In this paper, we used XRF 2D mapping because of the high resolution offered by this method to understand better Sr incorporation in pipefish otoliths. The underlying biological question is the diadromous life cycle of tropical freshwater pipefish. Sr XRF 2D mapping was recently used on the otoliths of two tropical freshwater species [7], *Microphis brachyurus* (Bleeker, 1854) and *Microphis nicoleae* Haÿ et al., 2023 [19]; it was shown that these two species navigate between water masses of different salinity during their life, a strong suggestion that they are amphidromous. In this paper, we further analysed *Microphis brachyurus* (Sri Lanka to French Polynesia) and *M. nicoleae* (Papua New Guinea and the Solomon Islands) and added two species, *Microphis torrentius* Jordan and Seale, 1906 (Samoa, Vanuatu and New Caledonia), and *Microphis cruentus* Dawson and Fourmanoir, 1981 (New Caledonian endemic). We used hyperfine 2D methods and developed analytical imaging processes to reveal otolith Sr incorporation patterns and to retrieve information on life history traits. 

## 2. Materials and Methods

### 2.1. Sample Preparation

**Figure 1:**
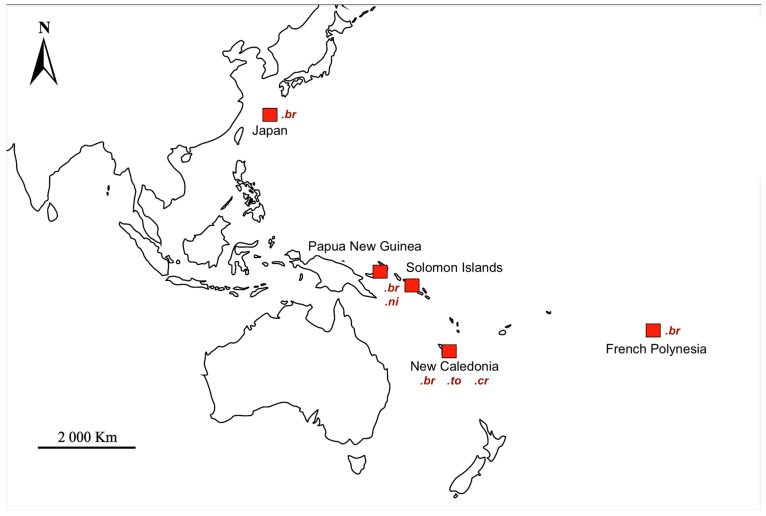
Collection sites in the Western and Central Pacific Ocean region (red squares). *.br*—*Microphis brachyurus*, .*ni*—*Microphis nicoleae*, *.to*—*Microphis torrentius*, *.cr Microphis cruentus*.

Otoliths were obtained from the Muséum national d’Histoire naturelle (MNHN, Paris, France) collections: fish were collected during field campaigns and authorization was given by the curators to retrieve the otoliths. Freshwater Syngnathidae were sampled from different locations in the Indo-Pacific realm and preserved in 95% ethanol, a preservation medium that has been shown not to hamper microchemical analysis [34]. We used a total of 32 specimens: 17 *Microphis brachyurus,* 8 *Microphis nicoleae*, 3 *Microphis torrentius* and 4 *Microphis cruentus*. The standard length of each individual was consistent with syngnathids in adulthood (Figure 1, Table 1).

The otoliths (*sagittae*) were removed from the saccules of the inner ear of each sample, thoroughly rinsed with MilliQ water and kept dry until they were individually embedded in epoxy resin (Araldite 2020, Escil, Chassieu, France). The embedded otolith was cut on either side of its core along the longest transverse axis using a low-speed diamond-bladed saw (Buehler, Leinfelden-Echterdingen, Germany) and ground-on carbide silicon abrasive discs of decreasing grain size (Escil, Chassieu, France). A slice less than 200 µm thick was obtained exposing a frontal section down to the core. The slice was checked by light microscopy.

Utmost care was taken to prevent impurity deposition on the sample, especially metal contamination, at every step of the process. The resin was also used for mechanical fixation of the sample to avoid movement during the beam time.

### 2.2. Synchrotron-Induced XRF Scanning and Data Acquisition

X-ray fluorescence spectrometry (XRF) was performed by scanning the full-size area of the otolith cross sections at the Nanoscopium Nanoprobe CX3-scanning spectromicroscopy beamline of Synchrotron Soleil (Saint-Aubin, France).

The beamline (approx. 155 m long) allows focusing high energy and coherent X-ray beam from a U18 X-ray source undulator down to 100 nanometres. The incident monochromatic photon beam was set to 16.2 keV energy and focused by a Kirckpatrick–Baez mirror. The multilength scale capability of the beamline allows for adjusting the resolution of the map from 1 × 1 down to 0.3 × 0.3 (H × V µm^2^) for our experiment [35]. The sample was mounted on an X and Z translation stage allowing displacement with a positioning accuracy down to 100 nm and with a travel range up to 1 cm. Two silicon drift detectors (SDD VITUS H50, KETEK GmbH, Munich, Germany) were mounted at 120° to the incident X-ray beam to measure the X-ray fluorescence emitted by the sample. 

Spot-by-spot data was collected under continuous scanning by using the available FLYSCAN mode at the beamline. In XRF, being a full-spectral technique, the signal was simultaneously collected from all elements that fluoresce under the experimental conditions. Quantitative reconstruction of the data was conducted by an in-house process of raw data optimization by reducing the noise, removing artefact signals and measuring the discrete energy intensities of the target elements [36].

Energy calibration, a crucial step for element identification, is performed in a standard way from each data detector apart by selecting at least two known X-ray lines [37] and specifying the energy confidence limits allowed to contain the characteristic X-ray peak for each element. Then, each element derivation of the XRF spectrum is obtained by summing the energy signals from all the channels over which the characteristic peak of this element extends.

The output dataset is a collection of 2D elemental distribution encoded in Tag Image File Format (.TIF). In this study, the samples were subjected to the same conditions to obtain an X-ray energy intensity distribution of calcium (Ca) and strontium (Sr) with a pixel size of 0.5 µm and a dwell time of 40 ms. The acquisition time ranged from 1 to 3 h depending on the size of the surface area.

Sr:Ca analysis between samples was deduced from Sr and Ca X-ray signal intensity ratio. In order to retrieve the actual Sr mass fraction in the CaCO_3_ matrix in each sample, a calibration process was performed using PyMca stand-alone open-source software, institutionally supported and updated by the European Synchrotron Radiation Facility (ESRF) for spectral analysis (PyMca 5.9.2 http://pymca.sourceforge.net/, accessed on 6 June 2024) [38]. The corrected mass fraction was then translated to a molar fraction of Sr:Ca.

### 2.3. Best Use of Synchrotron Facility Allocation Time

The access to synchrotron beam time is not commonplace, hence most of the time allotted at the synchrotron facility site was dedicated to beam time use for data acquisition. Alongside the incompressible scan time, the large amount of raw data generated is imported reduced, corrected and pre-processed within the time frame of the experiment through an in-house built workflow pipeline for on-site data viewing. Then, a data reconstruction process makes the data outputs portable through regular storage devices thanks to a user-friendly interface and in the ready-to-use format (.txt, .TIF). All of the further data treatment steps are then feasible by the researchers on standard working stations.

### 2.4. Sr:Ca Ratio 2D Raster

Ca and Sr XRF data in TIF format was loaded as images using Fiji (Fiji Is Just ImageJ) software, the collaborative open source with a self-update project for scientific image analysis (http://fiji.sc/cgi-bin/gitweb.cgi, accessed on 6 June 2024) [39]. Each 2D raster image is displayed scaled in grey values, by assigning the black colour to the pixels with the highest fluorescence intensity signal, white for the pixel with the lowest intensity, and scaling the in-between intensities accordingly. Nevertheless, the encoded pixel value still calls the actual energy intensity figure in any of the calculations made by image processing, provided the high dynamic range (32 bit) is maintained. Sr:Ca 2D rasters were obtained through image processing using the ‘divide function’ of the ‘image calculator’ package. The tool performs the arithmetic operations pixel by pixel between the two ‘images’.

The outcome is a 2D raster of Sr:Ca X-ray energy ratio map, which is then either tuned to pixel-by-pixel colour allocation or used as is for their value dataset. In each raster, the Sr:Ca values along with their spatialization by addressing their cartesian coordinates were exported and saved as .csv (comma-separated values) in order to analyse zonation and intensity frequency distribution.

### 2.5. Colour Map Tuning

#### ‘Red-to-Slate Grey’ Sr:Ca Ratio Permutation

Sr:Ca colour maps are displayed by assigning a colour to the pixel’s value following a home-designed ‘red-yellow-brown-cyan-slate grey’ palette for permutation (scale range provided by the inset in the figures). An in-house built lookup table (LUT) was set using the LUT tool of the Fiji package library. The rational colour permutation criteria were normalised using the range of Sr:Ca ratios for all samples. To do so, a normalization step was performed by setting manually a few square pixels in each image background to the maximum Sr:Ca ratio value retrieved in the whole sampling. Then, the in-house lookup table was applied to the image scaled in grey values. The colour maps obtained reflected the differences in intensity matching the Sr:Ca ratio range in the sample and provided a visualization of the phenotypic variations between the samples.

### 2.6. 3-3-2 RGB Image Conversion

The 3-3-2 RGB conversion process was applied to the Sr:Ca 2D rasters using the dedicated LUT tool of the Fiji software. Tabulation transforms the pixel value by addressing three times the value to red colour, three times to green and two times to blue. This mathematical transformation allows visualization of neighbouring information. The closer the adjoining Sr:Ca values are, the smoother the offset in the converted image is. This method provides an interesting addition to the basic Sr:Ca colour scale mapping as it can unmask the presence of subtle local gradients in the range of the raw values or, conversely, highlight sustained granular zones.

### 2.7. Post-Treatment Computation for Sr:Ca Ratio Frequency Distribution

As described in a previous study [7], zonal changes in Sr XRF signal intensity depict three concentric zones in the otoliths. This configuration is used to allocate each raster coordinate as belonging to one of three zones, which are named here—from the core to the periphery—Zone 1 (Z1), Zone 2 (Z2) and Zone 3 (Z3).

This was carried out by using Fiji software to adjust boundaries on the signal output intensity transitions and select the discrete areas of which the corresponding pixel cartesian coordinates (x,z) were allocated by means of specific transformation in the result (.csv) file and export process.

Then, the Sr:Ca values retrieved from the 2D raster were subjected to zone allocation by matrix superimposition of the values to the cartesian coordinate sets using basic tools of the collaborative open source R project available on the Comprehensive R Archive Network (https://cran.r-project.org/, accessed on 6 June 2024). All analyses were carried out with R version 4.2.3. Data tagged with allocation was then gathered and processed using ‘dplyr’ package and ‘summarise’ function in order to aggregate and summarise the Sr:Ca value frequencies. The figures were set up by running the ggplot2 package geom_area function for normalized frequencies (reported to surface area) above the 1% threshold value.

## 3. Results

For each otolith analysed, 2D maps show that Sr content is distributed in a concentric manner. The visualization of the Sr:Ca ratio reveals an integration of Sr within the otolith matrix in every growth direction.

### 3.1. Output Process

**Figure 2:**
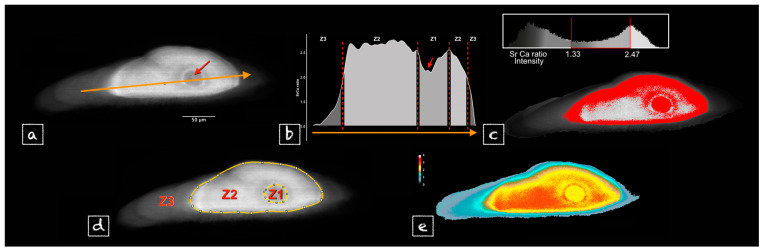
Workflow stages for the analysis of 2D Sr:Ca distribution. (**a**) Zonal pattern is highlighted by the XRF signal in the otolith section down to the core (red arrow); (**b**) Transition between zones are shown by steep slopes on the graph (red dotted lines) of Sr:Ca XRF signal ratio along a transect (orange transect) through the core (red arrow). (**c**) Three main zones can be defined by thresholding the Sr:Ca signal ratio values: Z1, quite circular zone of c. 20 to 30 μm centred around the core, then Z2 and Z3 from the core to the periphery, respectively. (**d**) Each pixel on the otolith section is assigned a tag addressing the zone it belongs to; it will then be used to extract the spatialized data. (**e**) Phenotypic variations between samples were shown using a colour gradient for the Sr:Ca ratio.

Figure 2 sketches the key steps of the data analysis. Prior to the analysis, we used the following starting postulates: (i) Sr is a major mineral component of the otolith and an element that marks water salinity, (ii) otoliths record time and chemical information and (iii) synchrotron-based 2D XRF is a semi-quantitative method. Figure 2a shows a zonal pattern on the Sr distribution image when Ca’s was smooth and uniform [7] indicating that the spatial distribution of the Sr:Ca ratio was strikingly driven by the Sr variation. The transect drawn from edge to edge through the core (Figure 2a) shows steep changes in the Sr:Ca ratio during the fish’s life (Figure 2b). Three main zones are highlighted by the use of Sr:Ca thresholding (Figure 2c): there is an outermost zone of lowest Sr:Ca intensity (called Z3); Z3 shows a clear break with Z2, of higher Sr:Ca intensity. Finally, the last zone identified, Z1, is a circular zone of c. 20 to 30 μm in diameter with the core at its centre (Figure 2d). Variations in Sr:Ca in the so-called zones were compared between samples by imaging (Figure 2e) and computation, allowing the interpretation of the results both in terms of life cycle hypothesis and Sr incorporation dynamics in the otolith.

### 3.2. Otolith Sr:Ca Distribution of Discrete Zones

**Figure 3:**
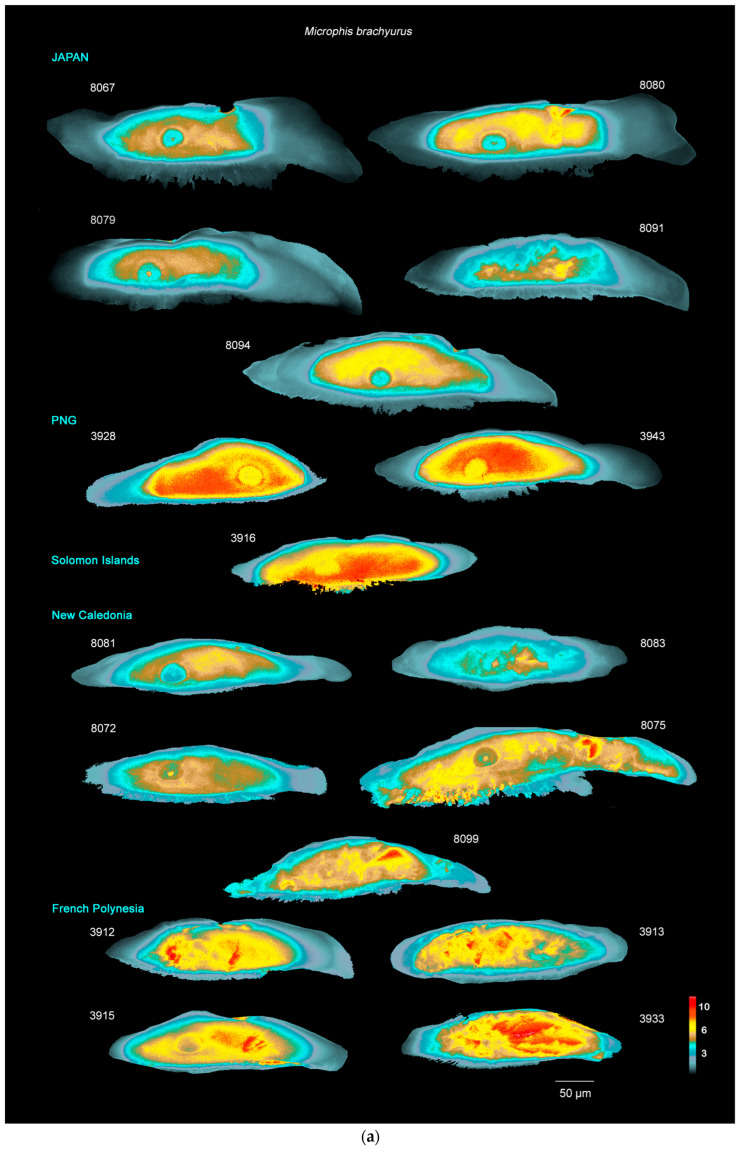

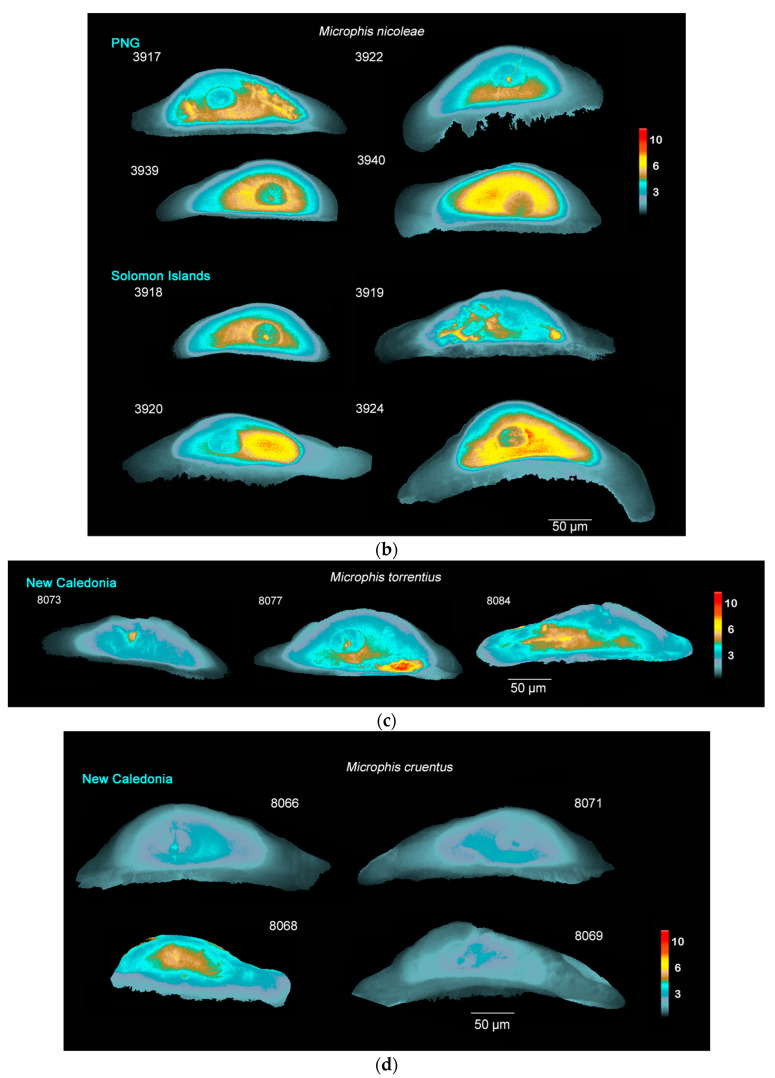
(**a**–**d**) Fine and global Sr:Ca ratio intensities highlighted in colour scale on the 2D rasters. (**a**). *M. brachyurus*—(**b**). *M. nicoleae*—(**c**). *M. torrentius*—(**d**). *M. cruentus.* (PNG: Papua New Guinea). Colour scale: Sr:Ca mmol·mol^−1^.

**Figure 4:**
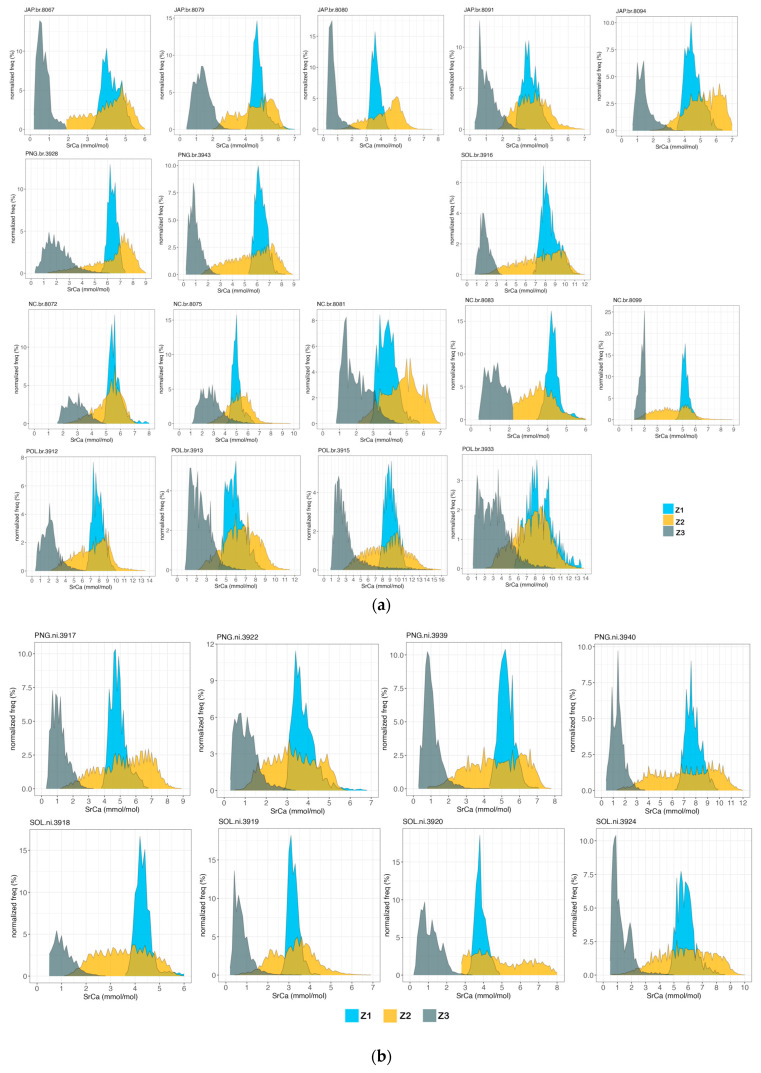

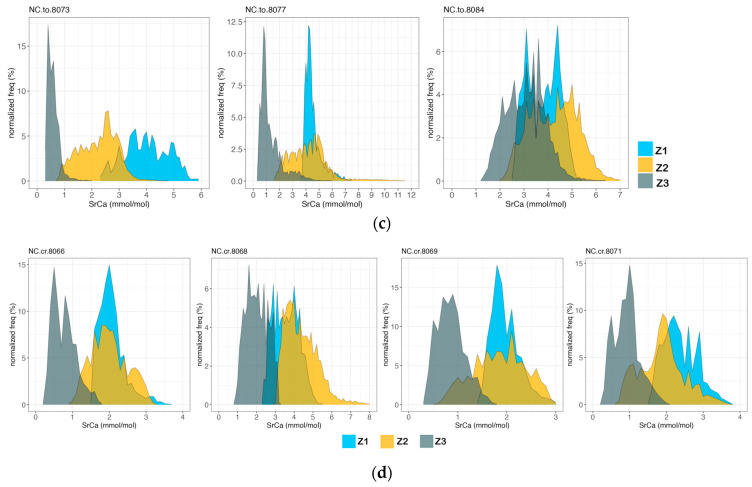
(**a**–**d)** Frequency distribution of the Sr:Ca molar ratio values computed from each individual 2D raster after the data has been assigned to the spatial zones of the otolith, Z1 (light blue), Z2 (orange) and Z3 (slate grey). The horizontal axis plots the Sr:Ca in molar concentration converted from XRF energy ratio values. Frequency (y axis) was normalized to the depicted zone surface (pixel unit). JAP: Japan, POL: French Polynesia, PNG: Papua New Guinea, SOL: Solomon Islands, NC: New Caledonia. (**a**). *.br*: *M. brachyurus*—(**b**). *.ni*. *M. nicoleae*—(**c**). *.to M. torrentius*—(**d**). *.cr*: *M. cruentus*.

Figure 3 represents the 2D Sr:Ca (mmol·mol^−1^) maps with the visualisation of the three concentric zones for each otolith. As observed with the 2D maps, over the zonal Sr:Ca pattern, the distribution of Sr:Ca ratios seems to be heterogeneous in the otolith. Figure 4 represents the frequencies of the Sr:Ca ratio in each zone defined in the otolith in order to characterize the variation in the elemental incorporation. The latter representation allows seeing the spread of values in each zone, and apart from a few individuals (see further in the results), Z3 is always clearly separated from the other two zones with homogeneously low Sr:Ca concentrations, consistent with a freshwater environment (around 2 mmol·mol^−1^) and a low spread of value centred on a peak. Z1 and Z2 always overlap, but Z1 shows a low spread of values centred on a peak unlike Z2, which shows a heterogeneous distribution, a very diffuse spread of values and no peak.

### 3.3. Environment Transition Signature in M. brachyurus and M. nicoleae

All specimens show a concentric Sr:Ca pattern consistent with transitions between environments of varying salinity (Figure 3a and Figure 4a; Figure 3b and Figure 4b). There is always a clear break between the outermost Z3 and Z2, with a very low Sr:Ca in Z3, consistent with life in freshwater (around 1 to 2 mmol·mol^−1^) and a Z2 area with Sr:Ca main maximum concentration around 5 to 9 mmol·mol^−1^, consistent with the marine environment. For most specimens, [Sr:Ca]_Z1_ < [Sr:Ca]_Z2_ >> [Sr:Ca]_Z3_, with a discrete innermost Z1 region (e.g., JAP 8094). This pattern will be called ‘classic pattern’ hereafter. Based on this classic pattern there are a number of noticeable variations: there are specimens for which [Sr:Ca]_Z1_ = [Sr:Ca]_Z2_ >> [Sr:Ca]_Z3_ (e.g., NC 8099 or POL 3912); several specimens (e.g., 8091, 3917 and 3922) show many areas in Z2 with a low salinity signature, but with the occurrence of patches of high concentrations (>6 mmol·mol^−1^) giving a marine signature, suggesting that these individuals with a ‘patchy’ Z2 migrated between environments of different salinities. The results suggest that these two species from all locations (Japan, PNG, Solomon Islands, New Caledonia and French Polynesia) are migrating species. The four *M. brachyurus* specimens from French Polynesia show a very heterogeneous Z2 with patches of very high concentration (up to about 16 mmol·mol^−1^; see specimen 3915), which do not concur with a concentric growth pattern. These specimens show the highest concentrations of Sr:Ca in Z2, compared to fish caught in other regions (maximum 7 mmol·mol^−1^ for specimens caught in Japan, 9 mmol·mol^−1^ in PNG, 12 mmol·mol^−1^ in the Solomon Islands and around 8 mmol·mol^−1^ in New Caledonia).

### 3.4. M. torrentius

For specimen 8073, Z1 signs for an environment under the influence of high salinity with Sr:Ca up to 6 mmol·mol^−1^. Z2 mode is around 3 mmol·mol^−1^ (freshwater) and Z3 is less than 1 mmol·mol^−1^. This specimen does not seem to migrate to the sea during the juvenile phase represented by Z2. The other *M. torrentius* specimens (8084, 8077) show a ‘patchy’ Z2 with only some areas of high Sr:Ca concentration (between 6 and 12 mmol·mol^−1^), testifying to a passage in the marine environment (Figure 3c and Figure 4c). 

### 3.5. M. cruentus

For this endemic species to New Caledonia (Figure 3d and Figure 4d), the three zones Z1, Z2 and Z3 are clearly visible, but the variability of Sr:Ca between the different areas in the otolith is less marked, with overlapping values for the three zones delimited in the otolith indicating that this species probably stays in water bodies of low salinity throughout its life. For all specimens except 8068, Sr:Ca concentrations remain low and representative of freshwater residency (<4 mmol·mol^−1^). Although heterogeneously distributed in Z2, 8068 shows an overall high Sr:Ca (mean 6 mmol·mol^−1^ for Z2), meaning that this fish may have passed through a marine environment during the juvenile growth phase.

### 3.6. Added Value of 3-3-2 RGB Image Conversion Method

**Figure 5:**
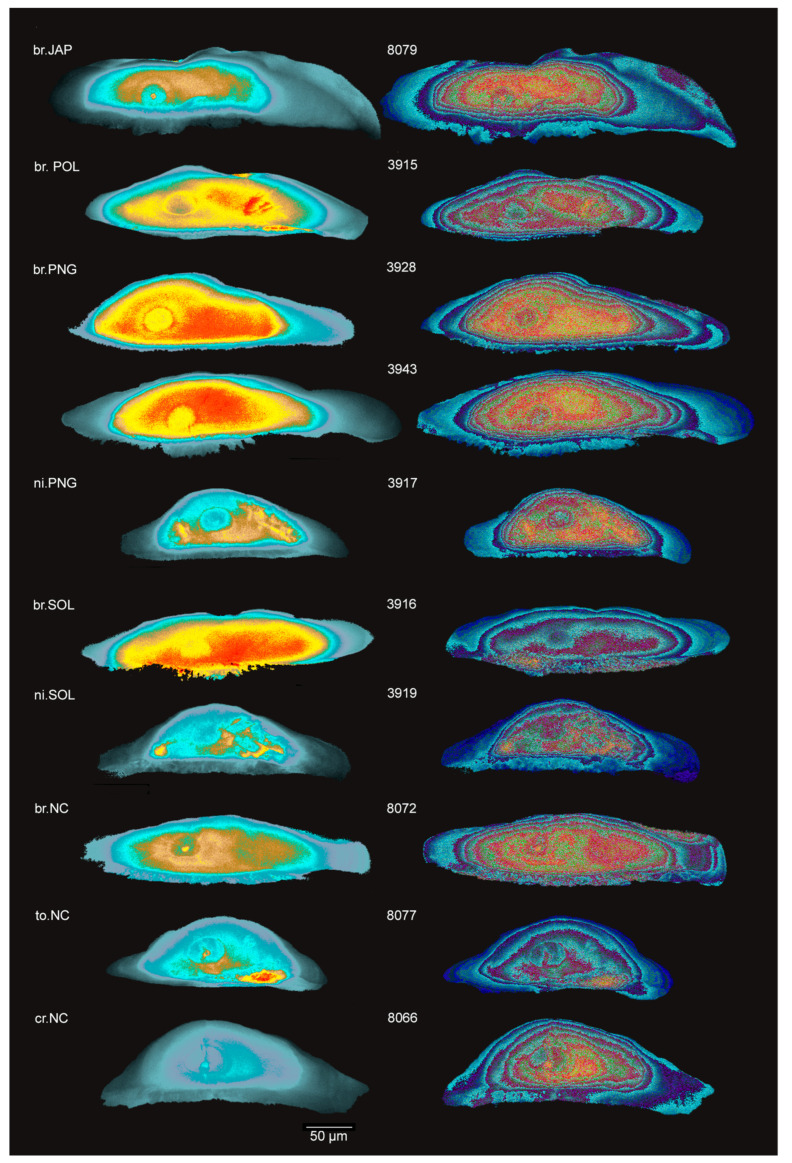
Colour-scaled Sr:Ca signal ratio raster pictures (**left side**) and their corresponding 3-3-2-RGB conversion (**right side**). The 3-3-2-RGB conversion method allows for enhanced offset information of each pixel with its immediate neighbours. *br: M. brachyurus*, *ni: M. nicoleae*, *to: M. torrentius*, *cr: M. cruentus*—JAP: Japan, POL: French Polynesia, PNG: Papua New Guinea, SOL: Solomon Islands, NC: New Caledonia—Scale bar: 50 µm—3-3-2-RGB conversion images were processed with filter for easier colour blindness reading.

The visualisation of Sr:Ca mathematically transformed on interleaved blocks of 3_square_pixels allows disentangling focal organisation in patches and local gradients and provides valuable additional information to the Sr:Ca maps (Figure 5). For all samples, Z2 shows a granular pattern but there are ring patterns at the Z2/Z3 boundary, which marks the return to freshwater after the marine phase. Interestingly, the ring zonation often extends in Z2. We can invoke that the ring patterns revealed by this method indicate stabling in estuaries or wandering at the river mouth. A likely explanation for the granular pattern could be the result of physiological and growth rate parameters overriding environmental chemistry at early times in the life cycle.

## 4. Discussion

In the present work, we analysed the Sr:Ca ratio of the otoliths of four species of freshwater pipefish. Diadromy was our entry point to study Sr incorporation in the otolith using 2D XRF mapping. Strontium (Sr) mapping revealed three alternating concentric zones consistent with the fish’s life stages (Figure 2, Figure 3, Figure 4 and Figure 5). This Sr integration is compatible with a circumperipheric continuous growth pattern as Sr is integrated in all growth directions. This Sr integration has already been shown for the European hake and the European sea bass: Sr:Ca does not show proximo-distal differences [26].

### 4.1. M. brachyurus and M. nicoleae: Amphidromous Species

The ‘classic pattern’ was the most common among *M. brachyurus* and *M. nicoleae*. It strongly suggests that these individuals are amphidromous: Z1 corresponds to hatching postulated to take place in a freshwater environment; Z2 with higher Sr:Ca corresponds to a marine phase (between 6 and 10 mmol·mol^−1^ and even higher) and Z3 with very low Sr concentration corresponds to life back in freshwater (<2 mmol·mol^−1^). These three life phases correspond to a typical amphidromous life cycle [5,7]. Adults live in freshwater; during field missions, adult fish are caught using an electric portative fishing device, which only functions in freshwater. Pipefish live upriver, as far up as the first impassable barrier, so often several hundred meters or several kilometres from the river mouth, far from the influence of salt water [40]. Mature adults are regularly observed in the rivers with the eggs borne by the adult male (pers. obs). The eggs hatch in freshwater and the juvenile phase, the phase where growth is very strong, takes place at sea. Juveniles then return to rivers where they colonise the adult habitat. Haÿ et al. [7] actually validated the amphidromous life cycle for the two species cited here and gave each zone a specific name: Z1 corresponds to hatching in freshwater (hFW), Z2 corresponds to the marine phase in sea water (SW) and Z3 corresponds to the adult phase in freshwater (AdFW). However, the Z1 region often exhibited Sr concentrations typical of environments of higher salinity than expected in freshwater (>5 mmol·mol^−1^). This could be explained by the fact that adults sometimes migrate to the estuary for the eggs to hatch closer to the sea, making it easier for juveniles to reach the marine environment.

*M. brachyurus* is the most widespread species studied in this paper, as is known from Sri Lanka to French Polynesia. Figure 3a and Figure 4a show that all the specimens studied are amphidromous, which is consistent with this species’ wide distribution. Marine Z2 signatures are quite different between specimens caught in the West Pacific Ocean (Japan, PNG, Solomon Islands and New Caledonia) and French Polynesia. These differences may be due to exposure to different environmental conditions (sea water signature may be different around French Polynesia than around Japan or New Caledonia) and/or to variations in the individual integration pathways. These different signatures may be used in the future to study migration routes for these amphidromous species.

### 4.2. New Caledonian Microphis: A Versatile Lifestyle?

*M. torrentius* is known from New Caledonia and Vanuatu, two close archipelagos, and Samoa further to the East. The three specimens studied in this paper were caught in New Caledonia. The maps and Sr:Ca profiles showed results comparable to what was analysed for *M. brachyurus* and *M. nicoleae* with two specimens (Figure 3c and Figure 4c: 8077 and 8084) with quite low Sr:Ca Z1 signature, a Z2 with patches of higher Sr:Ca signing for a marine environment and a Z3 of very low Sr:Ca, signing for freshwater. These two specimens are thought to be amphidromous. Specimen 8073, however, does not seem to migrate to sea during the juvenile growth phase. Indeed, Z1 shows a higher Sr:Ca (up to 6 mmol·mol^−1^) showing that the individual was under the influence of higher salinity at birth, probably as the adult migrated to the estuary during reproduction. Z2 then has a low Sr:Ca, signing for freshwater residency (around 2–3 mmol·mol^−1^) during the juvenile growth phase, and Z3 is typical of a freshwater environment (<1 mmol·mol^−1^). This specimen, 8073, stayed all its life in the river and did not undertake its juvenile development at sea. It did not have an amphidromous life cycle.

For *M. cruentus* (Figure 3d) the three concentric zones, Z1, Z2 and Z3, driven by Sr incorporation are clearly visible with Z1 and Z2 being higher in Sr concentration than Z3. *M. cruentus* 8068 has a ‘patchy’ Z2 with only some areas of high Sr:Ca concentration (up to 8 mmol·mol^−1^) (Figure 3d and Figure 4d), testifying to a passage in a marine environment during juvenile growth. For the other three *M. cruentus* specimens, the Sr:Ca molar ratio in all three zones reflects that of a freshwater environment (<4 mmol·mol^−1^) or at least a less pronounced transition to an environment of higher salinity. Maps shown in Figure 3d (NC 8066, 8071 and 8069) and graphs in Figure 4d suggest that these individuals are freshwater residents and that juvenile development took place in the river. 

These results suggest that some specimens of *M. torrentius* and *M. cruentus* do not seem to carry out their marine migration while others go to sea. Both species have a versatile lifestyle with a facultative amphidromous life cycle. Facultative amphidromy has been observed in other species, usually endemic or with a restricted geographic distribution. New Caledonia is known to harbour many endemic species, both plants and animals and both terrestrial and aquatic [41]. This is partially explained by the fact that New Caledonia is a particular geological formation with a metamorphic unit in the northeastern part of the island and peridotite nappes with an ultramafic nickel-rich soil in the southern part of the island [16]. Although the amphidromous life cycle is clear for the freshwater goby endemic to New Caledonia *Sicyopterus sarasini* Weber & Beaufort, 1915 [5], it is not obvious for the New Caledonian endemic goby *Protogobius attiti* Watson and Pöllabauer, 1988 [42]. The preference for an ultramafic substrate by New Caledonian species may explain the loss of an amphidromous life cycle. This observation is also relevant for the endemic *M. cruentus* for which facultative amphidromy is observed here. The number of specimens should be increased in order to state more precisely the amphidromous versatile modalities. Facultative amphidromy is known for fish and has been already observed in Hawaiian or Asian endemic species [43,44,45]. According to Closs et al. [46], the absence of complete migration between freshwater and marine environments in a few populations of amphidromous species (e.g., *Cottus, Rhinogobius* and *Galaxias*) could be explained by their specialization in the upstream habitats. Freshwater residency shows advantages: reduced mortality due to predation or the loss of larvae in ocean currents as well as reduced energy costs caused by the environmental transition [47]. The fragility of newly hatched pelagic individuals is a major cost of this life cycle, as they are unable to withstand starvation and other adverse physical and chemical conditions likely to be encountered during their downstream migration to reach their pelagic nursery habitat [48]. While some individuals may lose their ability to disperse, amphidromy is not lost on an evolutionary time scale, as it remains advantageous at the population or species levels for the colonisation of new habitats and for genetic mixing [47]. Therefore, variation in the amphidromous life cycle could be an adaption to particular environmental conditions. Since *M. torrentius* has a broader distribution than the endemic *M. cruentus* (New Caledonia, Vanuatu and Samoa), it was surprising to uncover the possibility of freshwater residency. Maybe it is caused by the particular conditions in New Caledonia. In any case, additional otoliths of this species from its entire distribution area should be analysed in order to quantify better the importance of facultative amphidromy for *M. torrentius*.

### 4.3. Advantages of 2D XRF Mapping When Addressing Biological Questions

The amphidromous life cycle and its versatility could have been uncovered using any 2D technic described in the introduction. 1D transect would even have allowed validating the amphidromous life cycle for otoliths with a ‘classic pattern’. But XRF methods allowed visualizing Sr integration within the calcified lattice at a hyperfine scale, finer than any of the other 2D methods, which led to surprising results. As our method mixes element quantification and 2D imaging, we were able to see that Sr concentration, especially in the Z2 area, is not at all homogeneously distributed. The spread of Sr:Ca values more or less important observed for Z2 (Figure 4) is consistent with the non-homogeneous integration of elements during the growth of the otolith during this phase representing an early life phase. Z2 has, for many specimens a patchy distribution, which does not fit with the otolith’s concentric growth even though hollow ring patterning can be unravelled underneath apparent gross features. This means that within a continuous growth band, heterogeneities occur. The heterogeneity of elemental distribution within the otolith matrix is greater than what is generally assumed [1,10,25,29]. Sr integration in the otolith may depend on the occurrence of vaterite inclusions, one of the three natural polymorphs of CaCO_3_ (calcite, aragonite and vaterite). Vaterite was shown to trap less Sr than aragonite [25,49,50]. In the present case, the four species studied were previously verified for the occurrence of CaCO_3_ polymorphs in their otoliths by using Raman spectroscopy. All the otoliths analysed only exhibited aragonite spectra (Supplementary Material Figure S1). As a result, we can hypothesize that this unusual pattern of Sr distribution is due to biomineralisation processes and the underlying control mechanisms. Most studies support that Sr is incorporated in equilibrium with environmental concentration [1,51,52,53], but that patchy distribution shows that Sr incorporation is also controlled by other additional factors, e.g., temperature, ontogeny, growth, metabolism, and this may particularly be true during the juvenile stage. Most studies find no effect of the temperature on Sr incorporation [53,54], but some experimental studies show that temperatures close to the species’ thermal tolerance limits may affect element uptake, transport in blood plasma or biomineralisation processes [55,56]. Temperatures in the rivers usually are around 23 to 25 °C (pers. obs). Sea surface temperatures around PNG, the Solomon Islands and French Polynesia are around 30 °C (https://www.ospo.noaa.gov, accessed on 22 February 2024). For amphidromous species, migrating from freshwater at hatching to the sea to spend their juvenile phase, the increase in water temperature of 5 to 8 °C may have a strong impact on individual metabolism and otolith growth via increased accretion [57]. In terms of endogenous and physiologically driven processes, ontogeny has a great impact on otolith growth. Although the environmental component in Sr uptake and incorporation is strong, it has been shown that ontogenetic changes alter fish growth and ion processing; therefore, important physiological and morphological changes in the fish’s life can affect Sr otolith incorporation, in addition to the strong environmental component [58,59]. There are very few studies on Sr incorporation independent of habitat use but some note that Sr can exhibit an ontogenetic decrease during larval or juvenile stages [60,61] while other studies show an increase in Sr with fish age [62,63]. For *Microphis* species, regardless of habitat use, Sr incorporation may be higher during the early life and juvenile stages due to physiological and genetic controls [64]. That may explain why even non-amphidromous individuals have higher Sr concentrations in Z1 and Z2 than in Z3.

Amphidromous species have to adapt twice to environmental changes affecting osmoregulation. The passage to marine life as juveniles is physiologically and metabolically challenging [65] and it impacts element incorporation within the otolith matrix, which may be reflected by the patchy pattern.

The rules of physico-chemistry may also be challenged by biological control [66]. For instance, the granular pattern could reflect the biological or genetic factors acting at a certain period in the life cycle on the growth directions of the otoliths and controlling their shape. The 3-3-2 RGB transformation allowed the enhancement of Sr incorporation information and visualisation of the internal growth dynamic of the mineral (Figure 4): the core zone Z1 and the first growth phase of Z2 have a granular aspect, which does not reflect the otolith growth by concentric accretion, and Sr incorporation is more discreet (mathematically speaking). Concentricity appears mid-Z2 or at the end of Z2, with a rhythmicity of Sr incorporation, particularly in the transition between Z2 and Z3. This transition is likely to reflect the transition from the sea to freshwater, and to a greater extent, the transition from a fast-growing juvenile phase to a slower growing adult phase (since this phenomenon is also seen on non amphidromous individuals). This shows that Sr incorporation during the early juvenile phase is under a biological driver and is influenced by ontogeny and growth.

The patchy effect can be very minimalistic in some specimens as patches of higher Sr concentration can only appear either on only one side of the otolith (towards the sulcus or towards the distal area) or on very small areas of Z2 and was uncovered only because hyperfine-scale XRF mapping was used (see Figure 3a *M. brachyurus* 8083; Figure 3b—*M. nicoleae* PNG 3922, 3917, SOL 3919, 3920; Figure 3c—*M. torrentius* NC 8077, NC 8084; Figure 3d—*M. cruentus* NC 8068). For these individuals, adding a second dimension to the analysis has proven to be a highly effective method. Typically, 1D analysis could have led to misinterpretation of the biology of these species as a transect, or points, chosen in a patchless area would have resulted in concluding to freshwater residency, while patches of high Sr are proof of environmental transition through a marine phase and show that the individual has undergone amphidromous migrations. We then conclude that all specimens analysed are amphidromous apart from three *M. cruentus* specimens, which remain their entire life in freshwater and *M. torrentius* specimens for which passage in marine environment is questionable.

## 5. Conclusions

Even though otolith microchemical composition is mainly driven by environmental elemental composition, biotic factors clearly act on element incorporation in the otolith lattice [1]. Synchrotron-based scanning X-ray fluorescence 2D high-resolution mapping allowed us to tackle global and hyperfine strontium heterogeneous distribution. It led to the discovery of plasticity in the freshwater pipefish life cycle as well as the trade-off expression between biological and chemical driving forces that govern element incorporation. This study illustrates the value of otolith chemistry to track fish movements, but caution must be taken against the routine interpretation of fish movement from otolith Sr:Ca variation under the assumption of a strict correlation with salinity. The analytical imaging process we developed and our results provide a strong basis for future works and prospects in fish biology and ecology. Fundamental knowledge of plasticity in the life cycle of amphidromous species has important implications in terms of biodiversity surveys, as varying life history traits are the motor of speciation and evolution. Furthermore, understanding life cycle modalities is crucial to categorize species distribution limits and to implement adapted conservation measures, especially when endemic species are at stake.

## Figures and Tables

**Table 1:** Sampling: species name, ID: field number; SL: Standard length in mm; collection location details (country, island, river); XRF otolith scan number; Museum national d’Histoire naturelle (MNHN, Paris, France) collection number.

Species	ID	SL	Sex	Country	Island	River	Scan	Collection Number
*M. brachyurus*	JP48	175.75	F	Japan	Okinawa	Genka	8067	MNHN-IC-2023-0276
*M. brachyurus*	JP44	187.81	F	Japan	Okinawa	Genka	8079	MNHN-IC-2021-0320
*M. brachyurus*	JP35	173.66	M	Japan	Okinawa	Teima	8080	MNHN-IC-2023-0274
*M. brachyurus*	JP32	165.12	F	Japan	Okinawa	Teima	8091	MNHN-IC-2023-0275
*M. brachyurus*	JP33	180.69	M	Japan	Okinawa	Teima	8094	MNHN-IC-2023-0275
*M. brachyurus*	17765	129.38	M	Papua New Guinea	New Britain	Rangihi	3928	MNHN-IC-2021-0318
*M. brachyurus*	17766	122.49	F	Papua New Guinea	New Britain	Rangihi	3943	MNHN-IC-2021-0318
*M. brachyurus*	19190	101.98	F	Solomon Islands	Kolombangara	Vaqe	3916	MNHN-IC-2021-0331
*M. brachyurus*	RTNC033	124.32	F	New Caledonia	Grande Terre	Creek Baie Nord	8072	MNHN-IC-2023-0273
*M. brachyurus*	RTNC117	168.60	M	New Caledonia	Grande Terre	Pwé hiit	8075	MNHN-IC-2023-0272
*M. brachyurus*	RTNC145	120.41	F	New Caledonia	Grande Terre	Garana	8081	MNHN-IC-2023-0277
*M. brachyurus*	RTNC139	105.57	F	New Caledonia	Grande Terre	Garana	8083	MNHN-IC-2021-0322
*M. brachyurus*	RTNC109	131.88	M	New Caledonia	Grande Terre	Pwé hiit	8099	MNHN-IC-2023-0272
*M. brachyurus*	PFV52	125.32	F	French Polynesia	Tahiti	Papenoo	3912	MNHN-IC-2023-0050
*M. brachyurus*	PFV06	105.02	F	French Polynesia	Tahiti	Papenoo	3913	MNHN-IC-2023-0051
*M. brachyurus*	PFV39	109.36	M	French Polynesia	Tahiti	Papenoo	3915	MNHN-IC-2023-0052
*M. brachyurus*	PFV42	104.15	F	French Polynesia	Tahiti	Papenoo	3933	MNHN-IC-2023-0053
*M. nicoleae*	19055	82.26	M	Papua New Guinea	New Britain	Walindi	3917	MNHN-IC-2023-0045
*M. nicoleae*	19183	69.41	F	Papua New Guinea	New Britain	Gavuvu	3939	MNHN-IC-2023-0446
*M. nicoleae*	19176	90.86	M	Papua New Guinea	New Britain	Gavuvu	3940	MNHN-IC-2023-0446
*M. nicoleae*	17693	93.66	M	Papua New Guinea	New Britain	Hoskins	3922	MNHN-IC-2021-0338
*M. nicoleae*	14962	64.37	F	Solomon Islands	Ranongga	Ovana	3918	MNHN-IC-2021-0336
*M. nicoleae*	14961	83.33	F	Solomon Islands	Ranongga	Ovana	3919	MNHN-IC-2021-0036
*M. nicoleae*	18253	97.65	M	Solomon Islands	Santa Isabel	Rakata	3920	MNHN-IC-2023-0047
*M. nicoleae*	18268	103.90	M	Solomon Islands	Santa Isabel	Rakata	3924	MNHN-IC-2023-0048
*M. torrentius*	RTNC119	84.82	F	New Caledonia	Grande Terre	Garana	8073	MNHN-IC-2021-0335
*M. torrentius*	RTNC118	89.69	M	New Caledonia	Grande Terre	Garana	8077	MNHN-IC-2021-0335
*M. torrentius*	RTNC114C	86.15	M	New Caledonia	Grande Terre	Pwé hiit	8084	MNHN-IC-2021-0334
*M. cruentus*	RTNC080	112	F	New Caledonia	Grande Terre	Carénage	8068	MNHN-IC-2021-0348
*M. cruentus*	RTNC107	149.66	M	New Caledonia	Grande Terre	Ouenghi	8066	MNHN-IC-2021-0349
*M. cruentus*	RTNC106	110.23	F	New Caledonia	Grande Terre	Ouenghi	8069	MNHN-IC-2021-0349
*M. cruentus*	RTNC108	168.60	F	New Caledonia	Grande Terre	Ouenghi	8071	MNHN-IC-2021-0349

## Data Availability

Data available at https://data.indores.fr/dataset.xhtml?persistentId=doi:10.48579/PRO/2REAXP, accessed on 6 June 2024.

## Supplementary Materials

The following supporting information can be downloaded at: https://www.mdpi.com/article/10.3390/biology13060446/s1, Figure S1: Otolith Raman analysis.
