# Supplementary material for: Travelling in Microphis (Teleostei: Syngnathidae) Otoliths with Two-Dimensional X-ray Fluorescence Maps: Twists and Turns on the Road to Strontium Incorporation
Source: Biology (Basel). 2024 Jun 18;13(6):446. doi: 10.3390/biology13060446 (PMC11201212; doi:10.3390/biology13060446)
Supplement: Supplementary file 1 [file biology-13-00446-s001.zip › biology-3038356-supplementary.pdf]

Supplementary Figure:

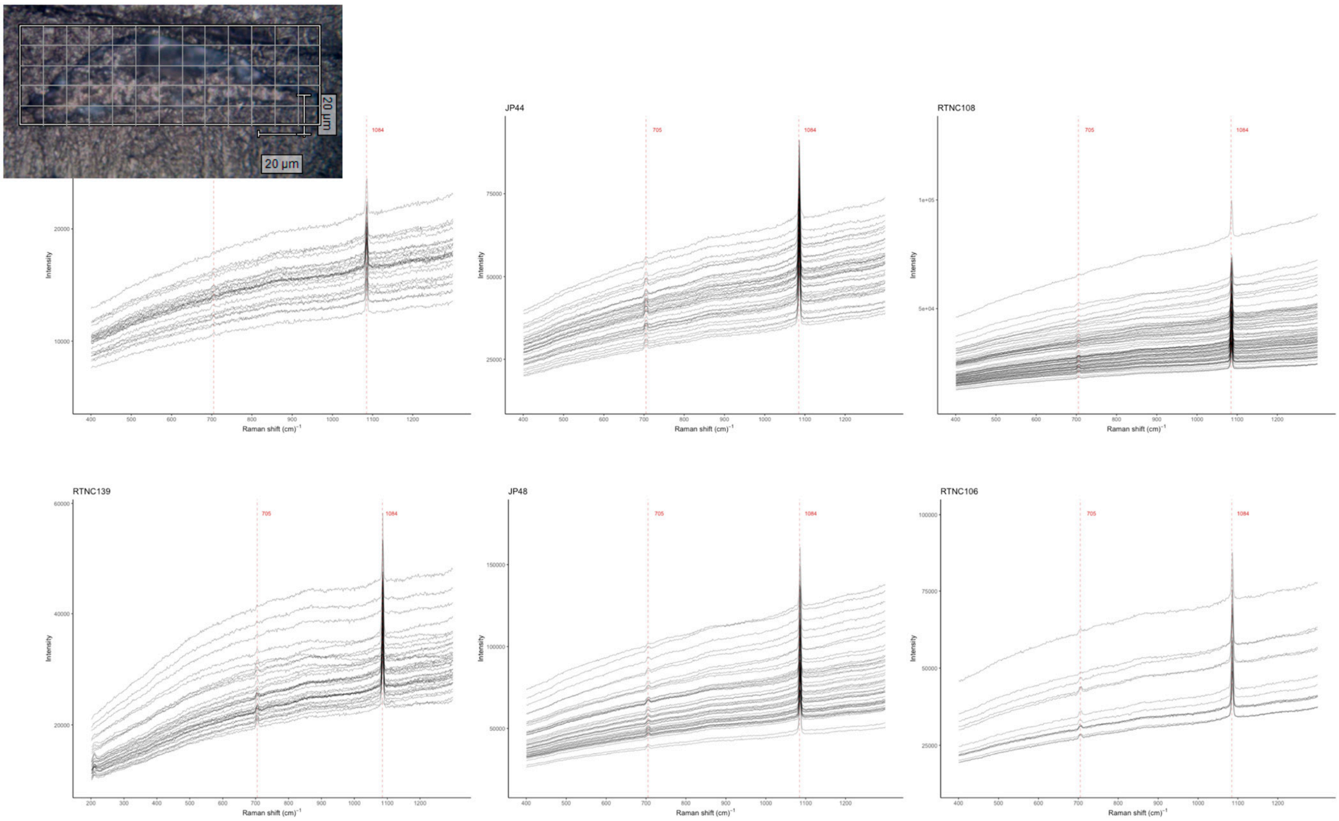

Figure S1: Otoliths Raman analysis: Analyses were performed using an inVia Raman microscope (Renishaw), 532 nm laser source set to 2.1 mW. The spectra were acquired with 5% power filter to avoid causing heating injury to the sample. Data was collected with a spectral resolution of 2 cm^{-1} every 10 µm step on maps designed to cover the sample surface (inset), the same that were previously subjected to XRF analysis. Each analysis was the co-addition of 3 spectra accumulated at up to 1s exposure. Raman resulting spectra show the unique characteristic aragonite shift signature in the otoliths (705 cm^{-1} and 1084 cm-1 characteristic bands).
